# Epidemiology of pathologic myopia in UK adults with high myopia

**DOI:** 10.1136/bjo-2024-326889

**Published:** 2025-08-25

**Authors:** Fabian Yii, Niall Strang, Miguel O Bernabeu, Baljean Dhillon, Tom MacGillivray, Ian J C MacCormick

**Affiliations:** 1 Robert O Curle Ophthalmology Suite, Institute for Regeneration and Repair, The University of Edinburgh, Edinburgh, UK; 2 Centre for Clinical Brain Sciences, Institute for Neuroscience and Cardiovascular Research, The University of Edinburgh, Edinburgh, UK; 3 Department of Vision Sciences, Glasgow Caledonian University, Glasgow, UK; 4 Centre for Medical Informatics, Usher Institute, The University of Edinburgh, Edinburgh, UK; 5 Princess Alexandra Eye Pavilion, NHS Lothian, Edinburgh, UK; 6 Institute for Adaptive and Neural Computation, School of Informatics, The University of Edinburgh, Edinburgh, UK

**Keywords:** Epidemiology, Risk Factors, Macula, Imaging, Retina

## Abstract

**Aims:**

To conduct the first cross-sectional epidemiological investigation of pathologic myopia (PM) in UK adults with high myopia.

**Methods:**

Fundus photographs of 3024 highly myopic eyes (spherical equivalent refraction (SER) ≤−5.00D) from 2000 randomly sampled adults (aged 40–70 years) in the UK Biobank were double graded by an ophthalmic reading centre using the Meta-analysis for Pathologic Myopia framework. Adjudication was performed by one of two retinal specialists. Multivariable mixed-effects logistic regression was used to explore potential risk factors and fundus biomarkers—initially adjusting for SER, age and sex, before including these and other variables with p<0.10 in a single model.

**Results:**

PM was present in 1138 of 3006 gradable fundus photographs, with 41.7% (95% CI 39.5% to 43.9%) of participants affected in at least one eye graded. Most eyes with PM exhibited diffuse chorioretinal atrophy (97.4%), while the more severe stages—patchy chorioretinal atrophy and macular atrophy—were observed in only 24 and 5 eyes, respectively. 13 eyes had ‘plus’ lesions or suspected staphyloma. Factors independently associated with increased odds of PM (all p<0.05) included decreasing SER (adjusted OR: 0.22, 95% CI 0.15 to 0.32), older age (2.20, 1.63 to 2.97), female sex (1.87, 1.12 to 3.12), lower deprivation (0.73, 0.56 to 0.94), white ethnicity (52.3, 17.3 to 158.3), lower retinal arteriovenous ratio (0.47, 0.37 to 0.58), increased retinal vascular complexity (4.60, 3.16 to 6.70) and a relatively horizontal disc orientation (2.98, 1.88 to 4.72). None of the explored modifiable lifestyle or health-related variables were associated with PM.

**Conclusions:**

PM prevalence is high among mid-life adults with high myopia in the UK Biobank, although most cases are relatively mild (diffuse chorioretinal atrophy). The only modifiable risk factor identified is myopia severity.

**WHAT IS ALREADY KNOWN ON THIS TOPIC**

- The prevalence of pathologic myopia (PM) is high among highly myopic adults in several population-based cohorts—with a pooled prevalence of 47.4%—but data outside Asia are very limited.

**WHAT THIS STUDY ADDS**

- The prevalence of PM is also high (around 40%) among highly myopic, mid-life adults in the UK Biobank, although most cases are relatively mild.
- Several retinal vascular and optic disc features are associated with increased odds of PM—independently of established risk factors such as myopia severity and age.

**HOW THIS STUDY MIGHT AFFECT RESEARCH, PRACTICE OR POLICY**

- The high prevalence of PM, coupled with its largely irreversible nature, underscores the need to prioritise myopia control—rather than just vision correction—as the ‘standard of care’ for myopic children/adolescents in the UK.
- Variations in fundus features, including subtle changes in retinal vasculature (eg, fractal dimension), could potentially aid in personalised risk stratification.

## Introduction

 While myopia has traditionally been regarded as a relatively benign vision disorder, there is growing recognition of its insidious implications for ocular health.^1^ One important myopic sequela is pathologic myopia (PM), which has, in recent years, been identified as the leading cause of irreversible blindness among adults in parts of Asia.^2^ In the UK, its impact was noted as early as seven decades ago in a report by Sorsby (cited in Duke-Elder’s celebrated *System of Ophthalmology*): following a review of 36 617 certificates of blindness issued between 1951 and 1954, PM was identified as the most common cause of legal blindness in English adults aged 40–60 years—although there was no standardised definition of PM at the time.^3 4^

Due to its largely irreversible and visually debilitating consequences, the global economic burden of PM is substantial, reaching approximately US$6 billion in 2015 based on conservative estimates.^5^ The estimated prevalence of PM in population-based studies (almost all conducted in Asia) ranges from 25.3% to 71.4% in highly myopic adults (spherical equivalent refraction (SER) ≤−5D or −6D), with a meta-analysed prevalence of 47.4%.^6 7^ However, there is a paucity of data on the epidemiology of PM in the UK. Knowledge about its modifiable risk factors and imaging biomarkers also remains limited.^8^ The UK Biobank is a large-scale, population-based cohort of mid-life adults recruited from 22 sites across the UK. With its extensive breadth and depth, the dataset provides a unique opportunity to capture a detailed snapshot of PM at the population level. Using this dataset, we aimed to conduct the first epidemiological investigation of PM in the UK, estimating its prevalence and risk factors among adults with high myopia (SER ≤−5D).

## Materials and methods

### Cohort description

Study participants were derived from the UK Biobank Eye and Vision subcohort, which comprised 68 508 participants who underwent an extensive range of assessments, including various ophthalmic tests from 2009 to 2010. Detailed description of the dataset is available elsewhere.^9^ Briefly, the ophthalmic assessments included 45° macula-centred colour fundus photography (3D OCT1000 Mark II, Topcon Corporation, Tokyo, Japan), autorefracto-keratometry without cycloplegia (RC-5000, Tomey, Nagoya, Japan), distance visual acuity (VA) assessment using a digital LogMAR chart (Precision Vision, La Salle, USA) and intraocular pressure (IOP) measurement using the Ocular Response Analyzer (Reichert Corporation, Philadelphia, USA).

**Figure 1:**
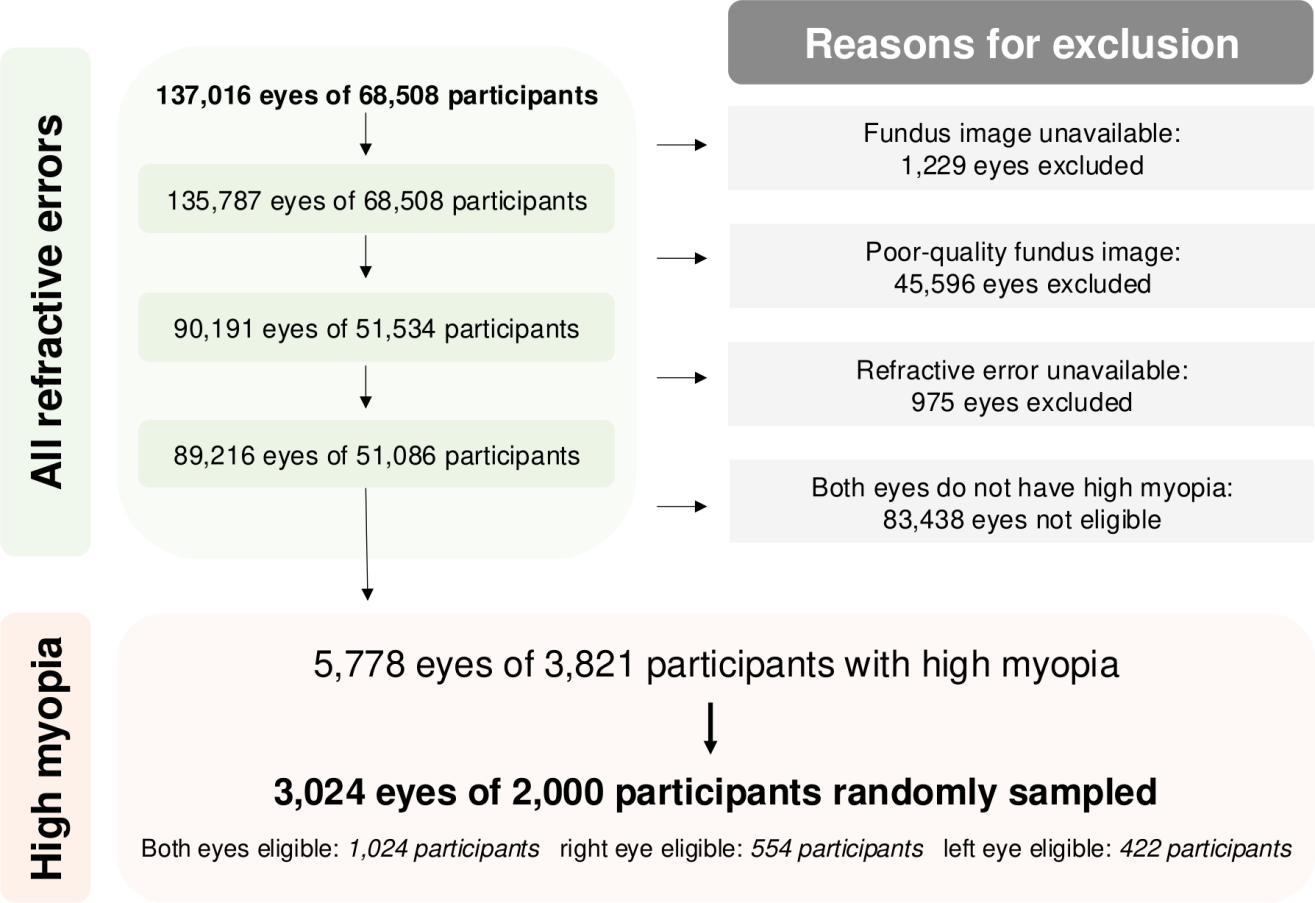
Flowchart providing an overview of the sample selection process, in which 2000 participants were randomly sampled from a population of 3821 participants with high myopia (spherical equivalent refraction <−5.00D) in at least one eye. Among those sampled, both eyes of 1204 participants, the right eye of 554 participants and the left eye of 422 participants had high myopia, making these eyes eligible for grading.

Figure 1 provides an overview of the sample selection process. After removing eyes without fundus photographs and those with ‘reject’ image quality, as determined using a validated deep learning model,^10^ 90 191 eyes of 51 534 participants remained. We further excluded eyes with missing refractive error and—among the participants that remained—3821 had high myopia in at least one eye (SER ≤−5.00D). Using an unstratified random sampling approach, we selected 2000 of these participants and graded the fundus photographs of 3024 eyes meeting the definition of high myopia outlined above. Online supplemental table 1 shows no significant differences in sociodemographic characteristics or SER between the 2000 sampled participants and the 1821 non-sampled participants.

### Pathologic myopia grading

Each fundus photograph was independently graded by two ophthalmic graders at the Belfast Ophthalmic Reading Centre (networcuk.com/Home/Belfast), using the META-PM framework.^11^ Myopic maculopathy, a key clinical manifestation of PM, was categorised into ‘no myopic maculopathy’ (M0), ‘fundus tessellation only’ (M1), ‘diffuse chorioretinal atrophy’ (M2), ‘patchy chorioretinal atrophy’ (M3) and ‘macular atrophy’ (M4). M2 was further divided into non-macular and macular subtypes.^2 12^ Examples of each category are available as online supplemental figure 1.

Three ‘plus’ lesions (lacquer cracks, myopic choroidal neovascularisation and Fuchs’ spot), which can coexist with any category of myopic maculopathy, are also part of the META-PM definition. We defined PM as the presence of any of the following: ≥M2 myopic maculopathy, ‘plus’ lesion(s) or suspected posterior staphyloma. One of two consultant retinal specialists adjudicated each instance of inter-grader disagreement. All graders, including the adjudicators, were masked to participant characteristics throughout the entire process.

### Risk factors

Several sociodemographic or lifestyle variables were explored as potential risk factors: (1) Townsend deprivation index (a numeric variable measuring relative material deprivation, where a more positive value indicated a greater degree of deprivation;^13^ (2) education level (post-secondary education vs secondary/lower education); (3) ethnicity (‘non-White’ vs ‘White’); (4) smoking status (never/previous vs current); (5) alcohol consumption (frequent vs infrequent, with ‘daily or almost daily’ as binary threshold) and (6) sleep duration (number of hours per day). Explored comorbidities or related variables (analysed as Boolean variables unless stated otherwise) included hypertension, cardiovascular disease, diabetes, glaucoma, body mass index (BMI) in kg/m^2^ (numeric), total cholesterol in mmol/L (numeric) and IOP in mm Hg (numeric). These are further detailed in online supplemental text 1.

### Fundus biomarkers

Several fundus imaging features were derived using methods detailed previously,^14^ focusing on dimensionless metrics to avoid issues related to camera telecentricity and ocular magnification.^15^ Vascular metrics included fractal dimension (FD; greater value suggested a more complex retinal vasculature), tortuosity (greater value indicated increased tortuosity), temporal arterial/venous concavity (greater value indicated a more inward course of the temporal artery/vein towards the fovea) and arteriovenous ratio (AVR; ratio of central retinal arteriolar equivalent to central retinal venous equivalent). These were all analysed as numeric variables.

The morphology of the optic disc (OD) was captured by measurements of its tilt (also known as ovality) and orientation. Tilt was the ratio of the major axis length to the minor axis length of the best-fitting ellipse, while orientation was the absolute angle between the horizontal axis of the image and the major axis of the OD. Both metrics were binarised for interpretability: a tilt value ≥1.3 suggested the OD was tilted,^16^,^19^ while an orientation angle ≥45° meant the OD was relatively vertically orientated. Recognising the challenges in accurately localising the fovea in cases of moderate/severe PM, metrics requiring foveal localisation were omitted.

### Statistical analysis

Multivariable logistic regression was used to explore potential risk factors and fundus biomarkers. Initially, separate models were fitted with each variable as the sole independent variable, while controlling for SER, age and sex. The p values for variables unrelated to SER and sociodemographic characteristics were adjusted to control the false discovery rate at 0.05.^20^ Subsequently, SER, age, sex and other variables with p<0.10 were included in a final model to assess their independent associations with PM. Sensitivity analyses were also performed by applying different binarisation thresholds for smoking status (never vs current/previous) and alcohol consumption (treating ‘daily or almost daily’ and the next highest consumption frequency, ‘three or four times a week’, as frequent consumption).

All models included random intercepts (individuals as random effects) to account for inter-eye correlation, fitted using the *glmer* function in R V.4.2.2 (R Core Team 2022, Vienna, Austria). Continuous (numeric) variables were all standardised to have zero mean and unit variance during model fitting to ensure model stability, as some variables were on very different scales. Multicollinearity was checked using variance inflation factor, treating 5 as the cut-off value.^21^ The source code is openly available at github.com/fyii200/UKbiobankPM

## Results

### Participant characteristics

18 of the 3024 assessed fundus photographs were ungradable due to image quality issues: 7 were unanimously rejected by both graders at the reading centre and 11 were further rejected during adjudication (examples are given in online supplemental figure 2), leaving 3006 eyes of 1994 participants for analysis. Table 1 and online supplemental table 2 present the summary statistics of key variables for the gradable and ungradable samples, respectively.

**Table 1:** Summary statistics of key variables (mean±SD shown for continuous variables)

Variable	*Overall* (*3006 eyes*)	*Normal* (*1868 eyes*)	*Pathologic* (*1138 eyes*)
Spherical equivalent refraction, D	−7.31±2.39	−6.90±1.82	−7.99±2.97
Age, years	54.7±7.7	54.5±7.8	56.0±7.5
Female, %	58.7	58.1	59.9
Townsend deprivation index	−0.97±2.90	−0.84±2.94	−1.20±2.85
> Secondary education, %	60.0	60.4	60.2
White ethnicity, %	91.1	87.6	97.7
Never or previously smoked, %	93.4	92.4	95.1
Alcohol: daily or almost daily, %	19.6	19.9	19.7
Sleep duration, hours per day	7.1±1.0	7.1±1.0	7.2±1.0
Hypertension, %	20.4	20.3	20.3
Cardiovascular disease, %	3.2	2.8	3.6
Diabetes, %	4.0	4.6	3.2
Body mass index, kg/m^2^	26.8±4.8	26.7±4.7	27.0±5.0
Total cholesterol, mmol/L	5.7±1.1	5.7±1.1	5.8±1.1
Glaucoma, %	2.4	2.4	2.4
Intraocular pressure, mm Hg	16.5±3.7	16.3±3.6	16.7±3.9
Vessel fractal dimension, %	1.44±0.07	1.44±0.07	1.45±0.06
Vessel tortuosity, %	0.69±0.04	0.69±0.04	0.69±0.04
Temporal arterial concavity	0.003±0.001	0.003±0.001	0.003±0.001
Temporal venous concavity	0.003±0.001	0.003±0.001	0.003±0.001
Retinal arteriovenous ratio	0.65±0.08	0.66±0.08	0.63±0.08
Tilted optic disc, %	2.4	1.7	3.6
Horizontally orientated optic disc, %	26.1	21.5	33.6

Eye-specific variables are summarised at the eye level, while participant-specific variables are summarised at the individual level.

White ethnicity accounted for 1817 (91.1%) of the 1994 participants included in the analysis. Most of the remaining 177 non-white participants were from Indian/Pakistani (n=56), Chinese (n=43) and Caribbean (n=42) backgrounds. The inter-grader agreement was high, with a weighted kappa coefficient of 0.80 and an overall agreement of 84%. Most adjudications were attributable to disagreements between M1 and non-macular M2 (50%), followed by non-macular M2 and macular M2 (28%), and M0 and M1 (9%).

### Disease prevalence

Overall, 1138 out of 3006 gradable fundus photographs showed signs of PM (37.9%, 95% CI 36.1% to 39.6%), with 41.7% (95% CI 39.5% to 43.9%) of participants affected in at least one eye graded. The majority (1107 eyes) exhibited the earliest stage of myopic maculopathy (M2), with the non-macular subtype being more prevalent than the macular subtype (648 eyes vs 459 eyes). Both patchy chorioretinal atrophy and macular atrophy were rare in this dataset, affecting only 24 (0.8%) and 5 (0.2%) eyes, respectively. Among eyes without myopic maculopathy, 1780 exhibited varying degrees of fundus tessellation.

12 eyes had ‘plus’ lesions, with the majority of eyes (n=10) showing concurrent myopic maculopathy (≥M2). Fuchs’ spot was found in two eyes with M1, one eye with non-macular M2 and one eye with M3. Lacquer cracks were present in five eyes with macular M2, one eye with non-macular M2 and one eye with M3. Only one eye was suspected to have posterior staphyloma, which coexisted with M3. The median (IQR) VA for eyes with M0, M1, non-macular M2, macular M2 and M3 was 0 (0.18), 0 (0.18), 0.02 (0.2), 0.06 (0.22) and 0.24 (0.5) logMAR, respectively. VA was available for only one (0.34 logMAR) out of the five eyes with M4 due to challenges in assessing VA in these eyes lacking good central fixation.

### Risk factors and fundus biomarkers

**Figure 2:**
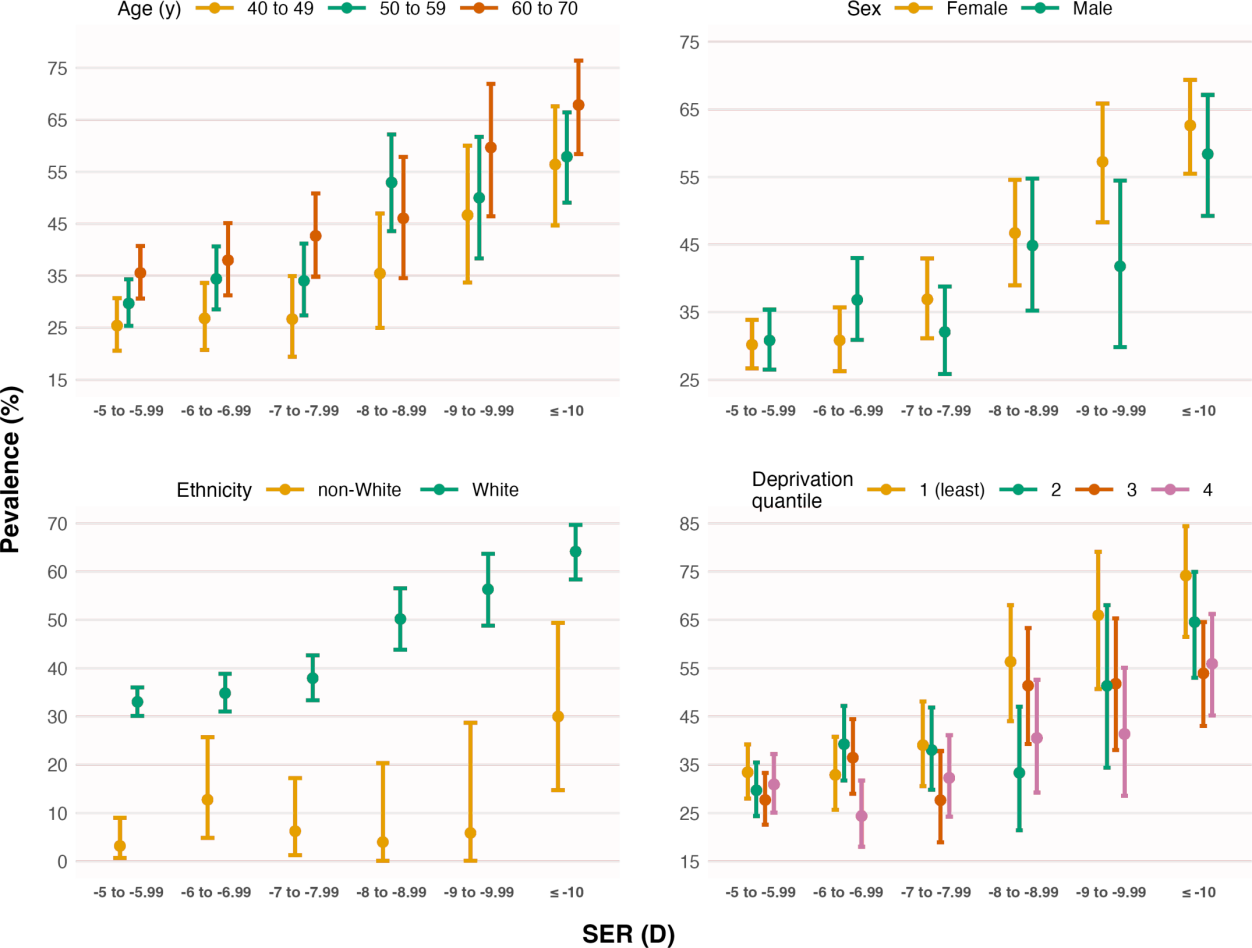
Prevalence (error bars represent the 95% CI) of pathologic myopia (PM) versus spherical equivalent refraction (SER), stratified by age, sex, ethnicity and Townsend deprivation index (divided into four quantiles for illustration purposes). While PM is present across all levels of SER, its prevalence increases non-linearly with increasing myopia. PM prevalence also increases with older age, female sex, white ethnicity and lower deprivation—trends that generally hold regardless of myopia severity.

Figures2  3 show the percentages of eyes with PM—stratified by sociodemographic variables and fundus features associated with PM in the final multivariable model (table 2). The variance inflation factor was low (≤2.2) for all variables in the final model, suggesting no major issues with multilinearity.

**Table 2:** Final multivariable logistic regression model with pathologic myopia (binary outcome) as the dependent variable, adjusting for spherical equivalent refraction (SER), age, sex and variables with p<0.10 from the initial models (adjusted for SER, age and sex; results shown in online supplemental table 3)

Variable	OR	95% CI	P value
SER	0.22	0.15 to 0.32	<0.001
Age	2.20	1.63 to 2.97	<0.001
Female	1.87	1.12 to 3.12	0.02
Townsend deprivation index	0.73	0.56 to 0.94	0.01
White ethnicity	52.3	17.3 to 158.3	<0.001
Vessel fractal dimension	4.60	3.16 to 6.70	<0.001
Retinal arteriovenous ratio	0.47	0.37 to 0.58	<0.001
Horizontally orientated disc	2.98	1.88 to 4.72	<0.001

All continuous variables were standardised to have zero mean and unit variance.

**Figure 3:**
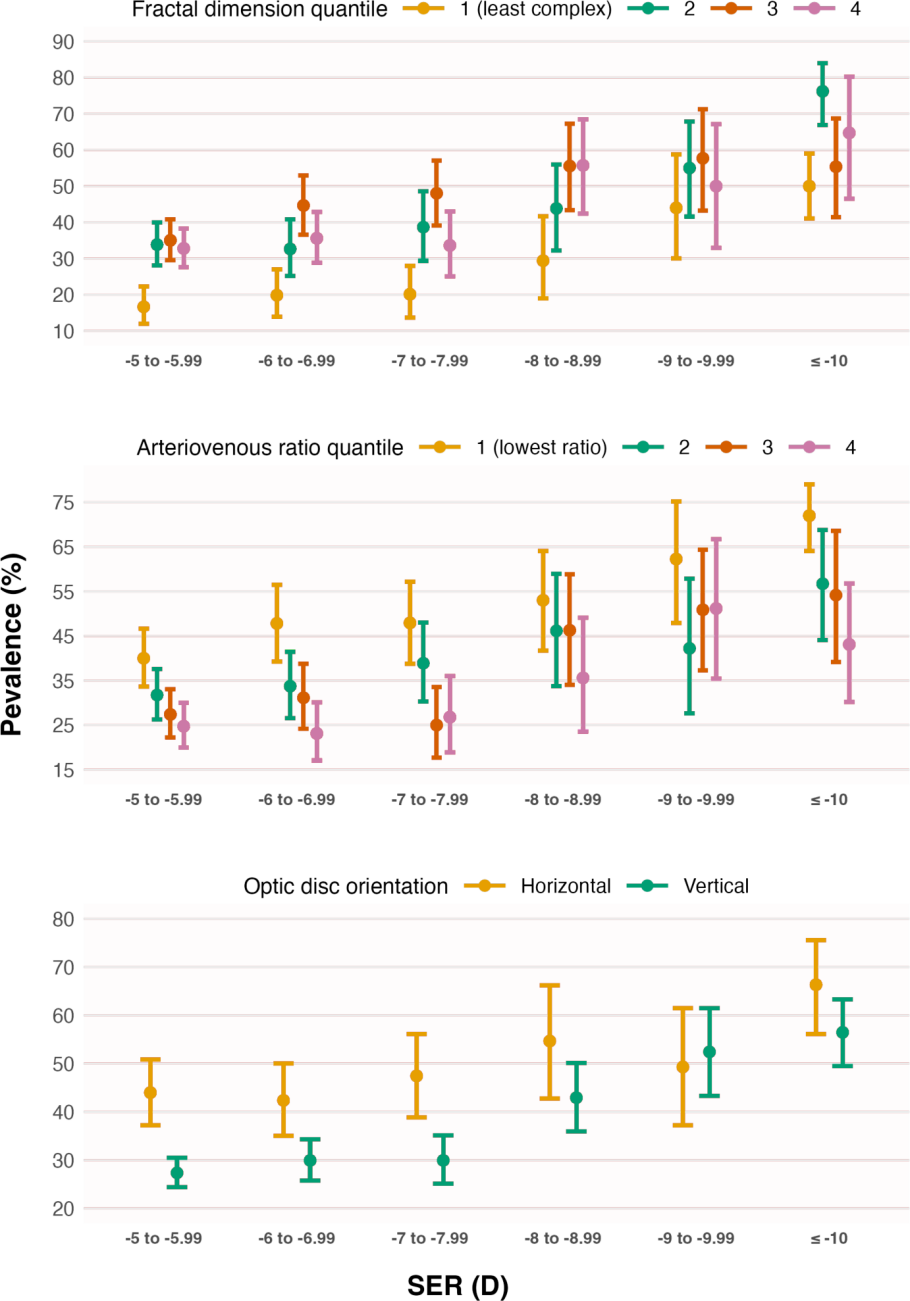
Prevalence (error bars represent the 95% CI) of pathologic myopia (PM) versus spherical equivalent refraction (SER), stratified by vessel fractal dimension (divided into four quantiles for illustration purposes), retinal arteriovenous ratio (divided into four quantiles for illustration purposes) and optic disc orientation (angle between horizontal axis of the image and major axis of the disc). PM prevalence increases with higher fractal dimension, lower arteriovenous ratio and a relatively horizontal optic disc orientation. These trends generally hold regardless of myopia severity, although they appear more pronounced at lower levels of myopia.

As seen in figure 2, PM prevalence was much lower (25.4%, 95% CI 20.6% to 30.7%) among younger participants aged 40–50 years with myopia >−6.00D, compared with older participants aged 60–70 years with myopia ≤−10.00D (67.9%, 95% CI 58.4% to 76.4%). The final model indicated a 4.6-fold increase in PM odds (reciprocal of the OR, 0.22) for every 1-SD decrease (more myopic) in SER, while the odds were estimated to be 2.2-fold higher for every 1-SD increase in age (table 2). Women had 1.9-fold higher odds of PM than men in the final model (table 2).

Additionally, white ethnicity was associated with increased odds of PM (table 2). Within the non-white ethnic group (overall prevalence: 10.7%), PM prevalence was 7.7% (95% CI 1.6% to 20.9%) among Indian/Pakistani participants, 3.3% (95% CI 0.1% to 17.2%) among Chinese participants and 3.7% (95% CI 0.1% to 19.0%) among Caribbean participants—lower than the 44.7% (95% CI 42.4% to 47.0%) observed in white participants. Participants from less deprived areas (more negative Townsend deprivation index) also had higher PM odds (table 2), with every 1-SD reduction in the index being associated with a 1.4-fold (reciprocal of 0.73) increase in odds.

Fundus features found to be independently associated with increased odds of PM included greater vessel FD (4.6-fold higher odds per 1-SD increase), lower AVR (2.1-fold higher odds per 1-SD decrease) and a relatively horizontally orientated OD (3.0-fold higher odds), as shown in table 2. While these imaging features were associated with PM irrespective of myopia severity, the effects appeared strongest in eyes with myopia between −5.00D and −8.00D (figure 3). Regression results for other non-significant variables are presented in online supplemental table 3, which includes all modifiable factors such as smoking status and alcohol consumption. Sensitivity analyses similarly indicated no independent association (p>0.05) of smoking status or alcohol consumption with PM odds.

## Discussion

The prevalence of PM among highly myopic adults in the UK Biobank is high, with slightly over 40% affected in at least one eye graded. However, most (97.4%) eyes with PM exhibited the earliest stage—diffuse chorioretinal atrophy (M2)—with the milder non-macular subtype (58.5%) being more prevalent than the macular subtype. Patchy chorioretinal atrophy and macular atrophy affected only 1% of all eyes with high myopia. Higher myopia, older age, female sex, white ethnicity, lower deprivation, higher vessel complexity, lower AVR and a relatively horizontal OD orientation were all independently associated with increased odds of PM. None of the modifiable lifestyle or health-related variables explored herein were associated with PM.

Among the few epidemiological studies on populations of European ancestry, PM prevalence was also found to be high among highly myopic (≤−6D) Dutch adults: 25.0%–24.5% and 35.2% among those aged 40–49, 50–59 and 60–69 years, respectively.^22^ In a German sample of 519 high myopes (SER ≤−6D) aged 35–74 years, the prevalence of PM was reported to be lower, around 10%.^23^ Differences in sample characteristics, such as a higher mean age (by 3.7 years) and higher proportion of women (58.7% vs 51.4%) in our sample, may partly explain the higher estimated prevalence in our study than Hopf *et al*.^23^ Despite variations in prevalence, all studies noted that most cases were relatively mild, with M2 being the most common presentation. In another older population-based study examining the prevalence of myopic retinopathy in predominantly white Australians aged ≥49 years, the prevalence was 25% for SER ≤−5D, with more than 50% of eyes with myopia above −9D affected.^24^ Likewise, among adults aged ≥40 years in the Beijing Eye Study, the prevalence of myopic retinopathy was found to be very high in 214 highly myopic eyes (≤6D): 40.4% for myopia between −6D and −7.99D and 89.6% for myopia ≤−10D.^25^ In the Singapore Epidemiology of Eye Diseases (SEED) cohort, PM was reported to affect 28.7% of 523 highly myopic (≤−5D) adults aged 40–80 years.^26^ Unlike the clear geographical variation in the prevalence of non-pathological myopia (much higher in East Asia),^27^ a high prevalence of PM among high myopes aged ≥40 years appears to be a feature common across geographical boundaries.

Older age and increasing myopia severity are two established PM risk factors.^8^ The deleterious impact of the age-related, progressive nature of PM is highlighted in recent work focusing on very old participants aged ≥85 years, where severe myopic maculopathy (≥M3) was observed in almost all eyes with high myopia.^28^ Notably, around 50% of participants with severe myopic maculopathy exhibited at least a moderate level of binocular visual impairment.^28^ These findings suggest an elevated risk of visual impairment for some participants in our study in later life—despite most disease presentations being mild at present (median VA ≤0.06 logMAR). The association between female sex and higher odds of PM, on the contrary, has not been consistently reported, with some studies^29^,^31^ finding evidence of an association but not others.^25 26^ A pooled analysis of population-based studies from across Asia (with covariate adjustment) provides strong cross-sectional evidence,^32^ although longitudinal evidence remains weak.^8^ This inconclusiveness notwithstanding, the general observation that women have a higher prevalence of PM fits with the well-known male-female health-survival paradox in epidemiology, where women tend to have more morbidities despite having a longer life expectancy on average.^33^

Consistent with previous studies,^25 29^ we found no association between education level (often used as proxy for socioeconomic status) and PM, although the pooled analysis mentioned above found evidence of an association between lower education and increased odds of PM.^32^ Using a more direct measure of socioeconomic status (Townsend deprivation index), we found that lower deprivation was independently associated with higher odds of PM, which runs counter to the general expectation that deprivation leads to higher risks of morbidities due to lower exposure to health-promoting lifestyle and behavioural factors. The significance and reason for this observation remain nebulous, but it may suggest that the ‘environment’ has a relatively limited influence on PM risk. This interpretation aligns with our findings that smoking, alcohol consumption, BMI and other modifiable variables had no influence on PM odds.

Similarly, the reasons for the significant difference in PM odds between white and non-white ethnicities remain unclear. Ethnic differences in myopia severity or sociodemographic variables (eg, deprivation level) cannot explain such a difference, as these were all adjusted for in our analysis. Given the lack of evidence that variables such as smoking status, alcohol consumption, BMI and total cholesterol were associated with PM odds in the present study, differences in environmental exposures also seem unlikely to be the cause. In the multi-ethnic SEED cohort, no ethnic difference was noted between Indians and Chinese/Malays after covariate adjustment. Caution should be exercised when interpreting our finding of an effect of ethnicity on PM, as unlike the SEED cohort, the UK Biobank was not designed as a multi-ethnic cohort (thus limiting its generalisability to ethnic minorities). Given the high prevalence of PM among highly myopic Chinese adults reported in the research mentioned above,^25 26^ the inordinately low prevalence of PM among Chinese high myopes in our study does not seem to support a potential role for genetics in explaining the observed ethnic difference in PM prevalence; rather, it might reflect a higher level of participation bias for ethnic minorities in the UK Biobank. That is, compared with white individuals, healthier-than-average ethnic minorities might have been more likely to participate.

Few studies have investigated imaging biomarkers in PM. Previously noted OD changes include disc enlargement, tilted disc (assessed qualitatively) and a less vertical disc orientation.^24 25^ However, it remains unclear from these studies whether such changes are *independent* of myopia severity due to the lack of adjustment for refractive error or axial length, as higher myopia is associated with a more tilted, larger and less vertically orientated OD.^14^ Indeed, in one study, an initially significant association between OD area and PM disappeared after controlling for myopia severity.^31^ Similarly, in our study, a significant association between tilted disc and PM (OR: 3.49, p=0.03) was observed in the absence of covariate adjustment, but this association disappeared after controlling for SER (online supplemental table 3)—suggesting that the univariable association between tilted disc and PM was primarily explained by the association between higher myopia and tilted disc. Previous work has also found that eyes with myopic retinopathy exhibit narrower central retinal arterioles and venules than myopic normal controls—even after controlling for refractive error—with arteriolar narrowing (−23.8 µm) appearing more pronounced than venular narrowing (−13.7 µm) on average.^34^ This aligns with our finding of a negative association between AVR and PM odds.

A higher FD—indicating a higher retinal vascular branching complexity (denser vascular network)—was found to be associated with PM odds in the present study, although Mao *et al*^35^ recently reported a decrease in retinal FD from 1.45 to 1.30 as myopic maculopathy increased from M0 to M4. In the absence of adjustment for myopia severity, however, their findings^35^ did not demonstrate whether FD had an independent association with PM and likely reflected FD changes due to increasing myopia from M0 to M1, since FD decreases with increasing myopia.^14^ Indeed, in our study, the mean FD for M0 was also higher (1.46) than for M3 (1.41) or M4 (1.35) when SER was not accounted for, reflecting increasing myopia severity from M0 to M4. After controlling for SER and other covariates in the final multivariable model, we found that PM was associated with higher, rather than lower, FD. It is unclear whether such an increase in vascular branching complexity is a direct reflection of alterations in retinal blood supply or an indirect reflection of the underlying choroidal vasculature becoming more visible en face due to the atrophy of the retinal pigment epithelium.^2^

Our study stands out for its depth (extensive geographical coverage) and breadth (vast amount of data collected) through the use of the UK Biobank database, as well as a significantly larger sample of high myopes (1994 analysed) compared with similar studies (≤626 high myopes).^22^,^2631^ However, the lack of ocular biometric data precludes further investigation into whether the associations of fundus features with PM were independent of axial length. The cross-sectional nature of the analysis also limits our ability to infer whether the changes in fundus features were precursors to, or consequences of, PM. Additionally, the UK Biobank is not fully representative of the UK’s mid-life population due to its volunteer-based nature and relatively low response rate (5.5%).^36^ Respondents were more likely to be older, female and socioeconomically advantaged than non-participants,^36^ characteristics associated with increased odds of PM (table 2). This implies that the estimated prevalence of PM is skewed towards individuals with these characteristics, likely resulting in some overestimation of prevalence compared with the general mid-life population with high myopia. Nevertheless, the study’s unique depth and breadth still provide a valuable, albeit imperfect, ‘first approximation’ of the scale of PM in the country. Future work analysing real-world data with linkages to routine health records could help us better understand the true scale of the disease.

## Conclusions

This is the first epidemiological study of PM in a UK population. The lack of association between PM and any of the explored modifiable variables—except for myopia severity—underscores the need to prioritise myopia prevention and control (rather than just vision correction) as the standard of care for children or adolescents, during which eye growth is modifiable.^37 38^ The significant associations of OD orientation, vessel FD and AVR with PM—independent of myopia severity and age—suggest that future longitudinal studies could explore whether these features might serve as useful adjuncts to established PM risk factors for facilitating personalised and early prediction of PM.

## Supplementary material

10.1136/bjo-2024-326889 online supplemental file 1

## Data Availability

Data may be obtained from a third party and are not publicly available.
