# Supplementary material for: Epidemiology of pathologic myopia in UK adults with high myopia
Source: Br J Ophthalmol. 2025 Aug 25;110(2):e326889. doi: 10.1136/bjo-2024-326889 (PMC12911562; doi:10.1136/bjo-2024-326889)
Supplement: online supplemental file 1 [file bjo-110-2-s001.pdf]

Supplementary Table 1

Comparison of demographic characteristics and spherical equivalent refraction (SER) between sampled participants (‘Participants’) and non-sampled participants (‘Non-participants’). Independent t-tests are used for continuous variables, while Chi-squared tests are used for categorical variables.

**Variable**	**Participants** *(n=2000)*	**Non-participants** *(n=1821)*	***P***
Age, y	55.1	54.7	0.12
Female sex, %	58.8	59.9	0.51
White ethnicity, %	8.9	8.5	0.71
> Secondary education, %	40.1	40.5	0.79
Townsend deprivation index	-0.97	-0.94	0.77
RE SER, D	-7.36	-7.22	0.11
LE SER, D	-7.27	-7.23	0.64

RE SER, right eye spherical equivalent refraction
LE SER, left eye spherical equivalent refraction

Supplementary Figure 1

Examples of eyes with different categories of myopic maculopathy: normal (M0), tessellation only (M1), non-macular diffuse chorioretinal atrophy (non-macular M2), macular diffuse chorioretinal atrophy (macular M2), patchy chorioretinal atrophy (M3) and macular atrophy (M4). Myopic maculopathy is defined as non-macular M2 or above (labels highlighted in red).

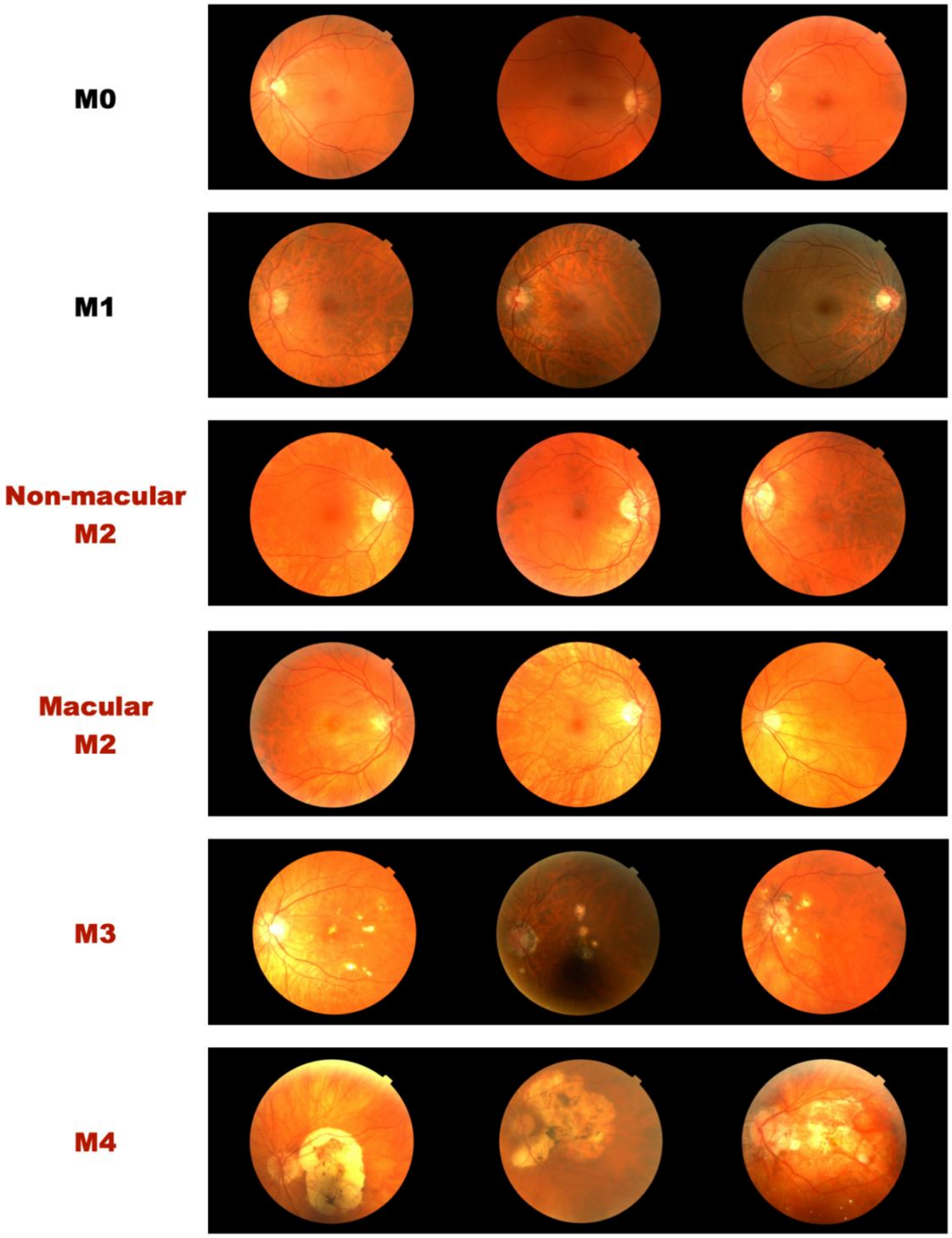

Supplementary Figure 2

Examples of fundus photographs that were not graded due to quality issues, including global overexposure (top left & top right), significant misalignment causing a large part of the macula to be hidden (bottom left) and underexposure obscuring the macula (top right & bottom right).

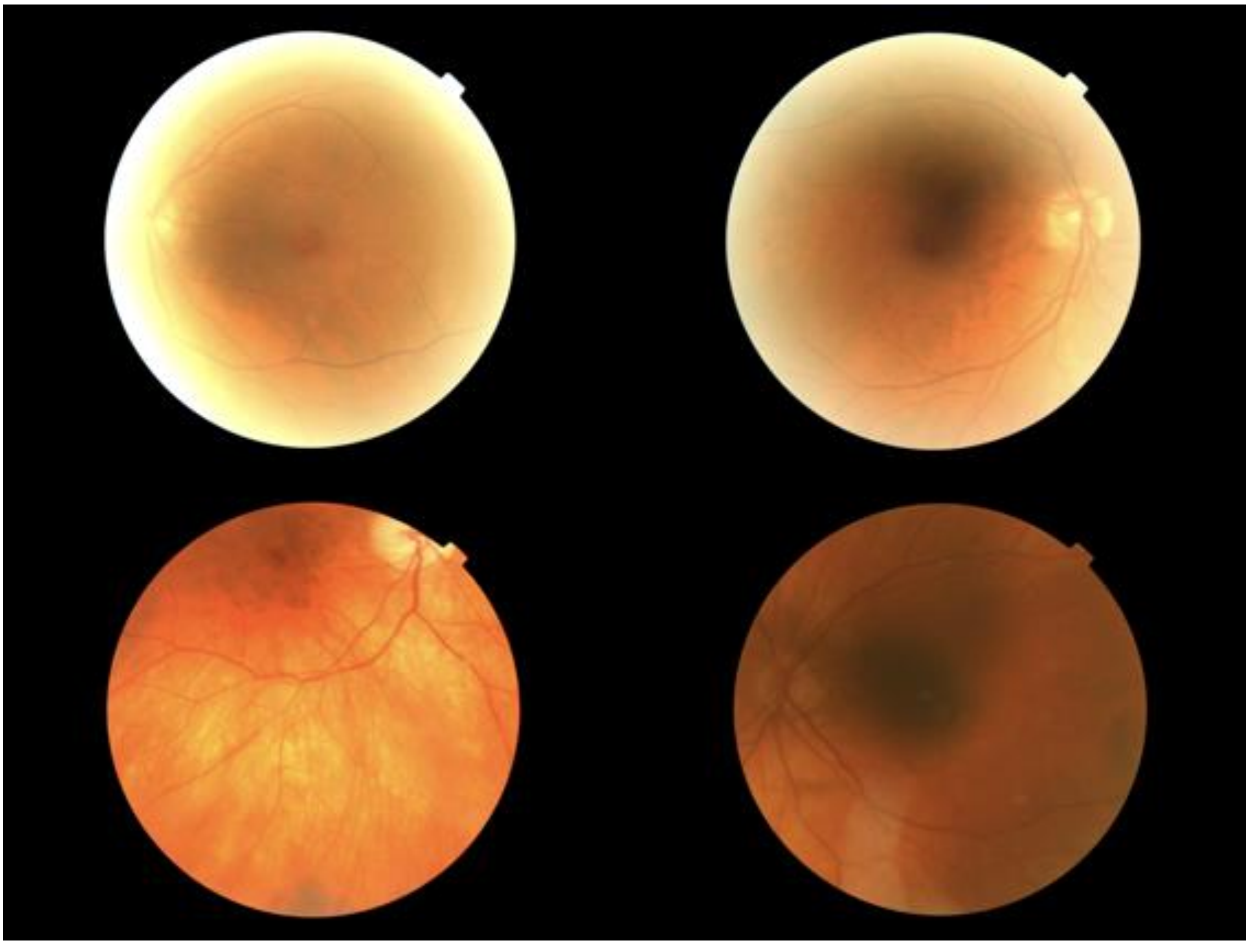

Supplementary Text 1

1. Hypertension: defined using linked healthcare data[1] or if either the systolic or diastolic blood pressure, measured with the Omron HEM-705IT (Omron Corporation, Kyoto, Japan), was equal to or exceeded 140mmHg or 90mmHg, respectively, on the day of assessment.
2. Cardiovascular disease: defined based on linked healthcare data[1] as a history of myocardial infarction, cardiomyopathy, ischaemic heart disease, cardiac arrest, heart failure, multiple valvular heart disease, atherosclerosis or any other heart condition.
3. Diabetes: defined based on linked healthcare data[1] or if random glucose, measured by hexokinase analysis on the Beckman Coulter AU5800 (Beckman Coulter, Brea, USA), exceeded 11.1mmol/L (equivalent to 200 mg/dl) on the day of assessment.
4. Glaucoma: defined based on linked healthcare data.[1]
5. Body mass index: derived from anthropometric measurements using standard scales (Seca GmbH, Hamburgh, Germany).
6. Total cholesterol: derived from CHO-POD analysis on the Beckman Coulter AU5800 (Beckman Coulter, Brea, USA).

[1] Described in detail at biobank.ndph.ox.ac.uk/ukb/ukb/docs/first_occurrences_outcomes.pdf

Supplementary Table 2

Summary statistics of key variables (mean ± standard deviation shown for continuous variables) for the 18 ungradable eyes. Eye-specific variables are summarised at the eye level, while participant-specific variables are summarised at the individual level.

Variable	Summary statistic
Spherical equivalent refraction, D	-8.34 ± 4.2
Age, y	60.5 ± 5.9
Female, %	72.2
Townsend deprivation index	-0.76 ± 3.30
> Secondary education, %	50.0
White ethnicity, %	100
Never or previously smoked, %	88.9
Alcohol: daily or almost daily, %	16.7
Sleep duration, hours per day	6.9 ± 1.1
Hypertension, %	28.6
Cardiovascular disease, %	0
Diabetes, %	5.6
Body mass index, kg/m^2	25.6 ± 4.1
Total cholesterol, mmol/L	6.2 ± 1.0
Glaucoma, %	0
Intraocular pressure, mmHg	16.0 ± 3.4

Supplementary Table 3

Logistic regression results from the initial models, with each variable adjusted for spherical equivalent refraction, age and sex. All continuous variables were standardised to have zero mean and unit variance.

Variable	Pathologic myopia	
	Odds ratio [95% CI]	P
Spherical equivalent refraction*	0.22 [0.12 to 0.40]	<0.001
Age[†]	1.74 [1.21 to 2.51]	<0.01
Female[‡]	1.30 [0.64 to 2.65]	0.47
Townsend deprivation index[§]	0.71 [0.49 to 1.02]	0.06
White ethnicity[§]	43.1 [17.0 to 109.7]	<0.001
Vessel fractal dimension[§]	6.44 [4.20 to 9.87]	<0.001
Retinal arteriovenous ratio[§]	0.59 [0.44 to 0.79]	0.003
Horizontally orientated disc[§]	2.65 [1.50 to 4.69]	0.004
> Secondary education[§]	0.94 [0.46 to 1.91]	0.85
Sleep duration[§]	1.03 [0.73 to 1.45]	0.92
Never or previously smoked [§]	1.94 [0.46 to 8.21]	0.92
Alcohol: daily or almost daily[§]	0.78 [0.32 to 1.93]	0.76
Hypertension[§]	0.86 [0.42 to 1.78]	0.92
Cardiovascular disease[§]	1.45 [0.18 to 11.51]	0.92
Diabetes[§]	0.33 [0.05 to 2.10]	0.70
Body mass index[§]	1.21 [0.85 to 1.73]	0.70
Total cholesterol[§]	1.06 [0.74 to 1.51]	0.92
Glaucoma[§]	0.60 [0.06 to 5.58]	0.92
Intraocular pressure[§]	1.14 [0.84 to 1.56]	0.76
Temporal arterial concavity[§]	1.04 [0.79 to 1.38]	0.92
Temporal venous concavity[§]	0.87 [0.67 to 1.12]	0.70
Vessel tortuosity[§]	1.01 [0.79 to 1.31]	0.92
Tilted optic disc[§]	2.44 [0.47 to 12.72]	0.70

**Adjusted for age and sex; [†]Adjusted for SER and sex; [‡]Adjusted for SER and age; [§]Adjusted for SER, age and sex*
